# Chemokine Transfer by Liver Sinusoidal Endothelial Cells Contributes to the Recruitment of CD4^+^ T Cells into the Murine Liver

**DOI:** 10.1371/journal.pone.0123867

**Published:** 2015-06-08

**Authors:** Katrin Neumann, Ulrike Erben, Nils Kruse, Katja Wechsung, Michael Schumann, Katja Klugewitz, Alexander Scheffold, Anja A. Kühl

**Affiliations:** 1 Department of Medicine I for Gastroenterology, Infectious Diseases and Rheumatology, Campus Benjamin Franklin, Charité—Universitätsmedizin Berlin, Berlin, Germany; 2 Research Center Immunosciences, Charité—Universitätsmedizin, Berlin, Germany; 3 Department of Cellular Immunology, Clinic for Rheumatology and Clinical Immunology, Charité—Universitätsmedizin, Berlin, Germany; 4 German Rheumatism Research Centre Berlin, an Institute of the Leibniz-Association, Berlin, Germany; Academic Medical Centre/University of Amsterdam, NETHERLANDS

## Abstract

Leukocyte adhesion and transmigration are central features governing immune surveillance and inflammatory reactions in body tissues. Within the liver sinusoids, chemokines initiate the first crucial step of T-cell migration into the hepatic tissue. We studied molecular mechanisms involved in endothelial chemokine supply during hepatic immune surveillance and liver inflammation and their impact on the recruitment of CD4^+^ T cells into the liver. In the murine model of Concanavalin A-induced T cell-mediated hepatitis, we showed that hepatic expression of the inflammatory CXC chemokine ligands (CXCL)9 and CXCL10 strongly increased whereas homeostatic CXCL12 significantly decreased. Consistently, CD4^+^ T cells expressing the CXC chemokine receptor (CXCR)3 accumulated within the inflamed liver tissue. In histology, CXCL9 was associated with liver sinusoidal endothelial cells (LSEC) which represent the first contact site for T-cell immigration into the liver. LSEC actively transferred basolaterally internalized CXCL12, CXCL9 and CXCL10 via clathrin-coated vesicles to CD4^+^ T cells leading to enhanced transmigration of CXCR4^+^ total CD4^+^ T cells and CXCR3^+^ effector/memory CD4^+^ T cells, respectively *in vitro*. LSEC-expressed CXCR4 mediated CXCL12 transport and blockage of endothelial CXCR4 inhibited CXCL12-dependent CD4^+^ T-cell transmigration. In contrast, CXCR3 was not involved in the endothelial transport of its ligands CXCL9 and CXCL10. The clathrin-specific inhibitor chlorpromazine blocked endothelial chemokine internalization and CD4^+^ T-cell transmigration **in vitro** as well as migration of CD4^+^ T cells into the inflamed liver *in vivo*. Moreover, hepatic accumulation of CXCR3^+^ CD4^+^ T cells during T cell-mediated hepatitis was strongly reduced after administration of chlorpromazine. These data demonstrate that LSEC actively provide perivascularly expressed homeostatic and inflammatory chemokines by CXCR4- and clathrin-dependent intracellular transport mechanisms thereby contributing to the hepatic recruitment of CD4^+^ T-cell populations during immune surveillance and liver inflammation.

## Introduction

Migration of leukocytes into tissues is central for immune surveillance and pathogen defence as well as for the pathogenesis of autoimmune diseases. Leukocyte recruitment from the circulation is a tightly regulated multistep process depending on specific interactions between adhesion molecules and chemokine receptors expressed by the leukocytes and their respective ligands on the vascular endothelial cells [1,2]. The model of transmigration across an endothelium involves several distinct steps. Endothelial selectins mediate initial tethering and rolling of circulating leukocytes whereas endothelium-bound chemokines trigger subsequent stable adhesion and transendothelial migration [3,4]. Transcytosis, a process by which molecules internalized at one plasma membrane of a polarized cell are transported via vesicles to the opposite membrane, is one assumed mechanism how perivascularly expressed chemokines reach the blood-endothelial interface for presentation [5,6].

Chemokines can be generally classified into inflammatory and homeostatic chemokines. Constitutively expressed homeostatic chemokines like the CXC chemokine ligand (CXCL)12 are involved in the control of physiological leukocyte recirculation and in immune surveillance. Inflammatory chemokine expression is induced in inflamed tissues by pro-inflammatory cytokines like interferon (IFN)-γ and consistently controls recruitment of effector cells determining the composition of inflammatory cell infiltrates and the outcome of local inflammation [7,8]. CXCL9 and CXCL10 belong to the group of inflammatory chemokines binding to the common CXC chemokine receptor (CXCR)3 [9] and are critical for the recruitment of effector T cells in various liver diseases [10–13].

Circulating T cells pass the liver via the sinusoids, a network of small capillaries lined by liver sinusoidal endothelial cells (LSEC). The sinusoidal endothelium is a major route for T-cell entry into the liver parenchyma from where recruited cells can subsequently migrate to sites of inflammation [14,15]. T-cell rolling is greatly attenuated in the liver probably due to low levels of shear stress in hepatic sinusoids. The small luminal diameter of the sinusoids in combination with the slow hepatic blood flow in itself is sufficient to allow prolonged contact for the first interactions between lymphocytes and LSEC [16,17]. Thus, endothelial chemokine supply may be particularly important for T-cell recruitment into the liver.

We previously showed that LSEC promote chemokine-dependent CD4+ T-cell transmigration *in vitro* [18]. In the present study, we addressed molecular mechanisms involved in LSEC-mediated chemokine supply in more detail and analyzed their contribution to the hepatic recruitment of CD4+ T cells during immune surveillance and liver inflammation.

## Material and Methods

### Mice

C57BL/6 mice were obtained from the Charité animal facility (Berlin, Germany) or Charles River (Wilmington, MA). CXCR3-/- mice [11] were kindly provided by PD Dr. Uta Höpken (Department of Tumor Genetics and Immunogenetics, Max-Delbrück-Center for Molecular Medicine, Berlin, Germany). All mouse experiments were conducted according to the German animal protection laws (Landesamt für Gesundheit und Soziales, Berlin, Germany; G 0022/09, G 0336/08, T 0183/07) with approval from the Charité—Universitätsmedizin ethical committee. All mice received humane care according to the national guidelines.

### Cell isolation

To isolate non-parenchymal cells (NPC), livers were perfused *in situ* with digestion medium containing collagenase IV (Sigma-Aldrich, Steinheim, Germany) injected into the portal vein, excised and further incubated in the digestion medium. To eliminate parenchymal cells, the single-cell suspension was subjected to a one-step density gradient centrifugation with 26% Nycodenz (Progen Biotechnik, Heidelberg, Germany). LSEC were isolated from NPC by magnetic cell sorting using anti-CD146 antibody (ME-9F1; BioLegend, Fell, Germany) as previously described [19]. LSEC adhered over night and were subsequently washed in order to remove non-adherent cells resulting in a purity of higher than 99% [20]. For transmigration assays, CD4+ T cells were isolated from spleen and lymph nodes using anti-CD4 MicroBeads (Miltenyi Biotec, Bergisch Gladbach, Germany) to a purity of at least 95%. Untouched CD4+ T cells used for homing assays were isolated by CD4+ T Cell Isolation Kit (Miltenyi Biotec) according to the manufacturer’s instruction to a purity of at least 95%.

### FACS analysis

NPC were stained with anti-CD4 (RM4-5), anti-CD90.2 (53–2.1), anti-CD69 (H1.2F3; all BD Biosciences, Heidelberg, Germany) or anti-CXCR3 antibody (CXCR3-173; eBiosciences, San Diego, CA). For detection of cytokine-expressing cells, NPC were re-stimulated with phorbol myristate acetate (10 ng/ml) and ionomycin (500 ng/ml) for 4 h with the addition of brefeldin A (10 μg/ml; all Sigma-Aldrich) after 60 min. Cells were fixed with 2% paraformaldehyde. After permeabilization using 0.5% saponin, NPC were stained with anti-IFN-γ antibody (XMG1.2; BD Biosciences). Unspecific binding was blocked with rat immunoglobulin (Dianova, Hamburg, Germany) and anti-CD16/32 antibody (93; BioLegend). LSEC were incubated with AlexaFluor 647-labeled CXCL10 or CXCL12 (both 10 nM; Almac, Craigavon, UK). LSEC were treated with chlorpromazine (CPZ; 30 μM), nystatin (10 μM) or filipin (15 μM) for 10 min and AMD3100 (10 μM; all Sigma-Aldrich) for 60 min, washed and further incubated with the chemokines for 60 min. Data were acquired using a FACS Canto II (BD Biosciences) and analyzed by the FlowJo software (Tree Star, Ashland, OR).

### *In vitro* activation of LSEC

*Ex vivo* isolated LSEC were cultured in the presence of tumor necrosis factor (TNF)-α (10 ng/ml; ImmunoTools, Friesoythe, Germany) and IFN-γ (20 ng/ml; R&D Systems, Wiesbaden, Germany) for 24 h. Adhered cells were detached by accutase treatment (Sigma-Aldrich).

### Transfection of LSEC

The expression vector Clathrin-LCa-EYFP (Addgene plasmid 21741) was provided by Chen Chen (Department of Chemistry and Chemical Biology, Harvard University, Cambridge, MA) [21]; CAV1-mEGFP (Addgene plasmid 27704) by Arnold Hayer (ETH Zurich, Institute of Biochemistry, Zurich, Switzerland) [22]. *Ex vivo* isolated LSEC (1x106) were re-suspended in 100 μl electroporation buffer (90 mM phosphate buffer, pH 7.2, 10 mM MgCl2, 50 mM glucose) before 4 μg plasmid DNA was added. Using an electroporation cuvette (2 mm gap; Lonza, Cologne, Germany) in the ELPorator 1000 device [23], LSEC were subjected to continuously combined high voltage (400 V/400 μs) and low voltage (150 V/20 ms) pulses. Immediately after pulse application, LSEC were transferred into pre-warmed Dulbecco's Modified Eagle Medium (Life Technologies, Carlsbad, CA) containing 10% fetal calf serum and were incubated at 37°C for 48 h.

### Real-time quantitative RT-PCR analysis

Total RNA was isolated from liver samples or LSEC by single-phase organic extraction (RNAPure; Peqlab, Erlangen, Germany) and 100 ng RNA was reversely transcribed into cDNA using the High-Capacity cDNA Reverse Transcription Kit (Applied Biosystems, Darmstadt, Germany). Quantitative PCR was performed using a TaqMan Universal Master Kit and exon-spanning, gene-specific assays (CXCL9, Mm00434946_m1; CXCL10, Mm00445235_m1; CXCL12, Mm00445553_m1; CXCR4, Mm01292123_m1; CXCR3, Mm00438259_m1; GAPDH, Mm03302249_g1; Applied Biosystems, Darmstadt, Germany) in a StepOne Plus real-time PCR system (Applied Biosystems). Specific chemokine and chemokine receptor mRNA expression was quantified in relation to Glyceraldehyde 3-phosphate dehydrogenase (GAPDH) as the housekeeping gene using the ΔΔCT method [24].

### Transmigration assay

LSEC were cultured overnight on gelatine-coated transwell membranes with a 5 μm pore size (Corning, Sigma-Aldrich) to form confluent cell layers [18]. LSEC were treated with the specific clathrin inhibitor CPZ (30 μM) [25], the caveolae-specific inhibitors nystatin (10 μM) or filipin (15 μM) [26] or the CXCR4 antagonist AMD3100 (10 μM) [27] added to the lower chamber of the transwell for 10 min (CPZ, nystatin, filipin) or 60 min (AMD3100). After washing, LSEC layers were pre-incubated with the chemokine (CXCL12, 50 nM; CXCL9, CXCL10, both 100 nM; all R&D Systems) applied to the lower chamber of the transwell for 120 min. After removal of the chemokine, 5x105 total CD4+ T cells were added to the upper chamber of the transwell and were allowed to transmigrate across the LSEC layer for 90 min. Transmigrated cells from the lower chamber or from the input were mixed with Fluoresbrite beads (Polysciences, Eppelheim, Germany), stained with anti-CD4 (GK1.5) and anti-CD45RB antibody (16A; both BD Biosciences) and analyzed by flow cytometry. Absolute cell numbers were determined by gating on CD4+ for total or CD45RBlow CD4+ for effector/memory CD4+ T cells in relation to defined numbers of beads.

### Confocal laser scanning microscopy

LSEC were cultured overnight on transwell inserts. AlexaFluor 647-labeled CXCL12 and CXCL10 (both 50 nM) or AlexaFluor 488-labeled acetylated-low density lipoprotein (AcLDL; 10 μg/ml; Life Technologies) were added to the lower chamber of the transwell for indicated times. Subsequently, LSEC were fixed with 4% paraformaldehyde (Sigma-Aldrich) and stained with mouse anti-clathrin (3F133), mouse anti-caveolin-1 (7C8; both Santa Cruz Biotechnology, Santa Cruz, CA), rabbit anti-early endosome antigen (EEA)1 (C45B10; Cell Signaling Technology, Danvers, MA), rabbit anti-green fluorescent protein (GFP), rabbit anti-transferrin receptor (TfR) and rat anti-lysosomal-associated membrane protein (LAMP)-1 (1D4B; all Abcam, Cambridge, UK) antibody followed by AlexaFluor 488-conjugated anti-mouse, AlexaFluor 647-labeled anti-rat, AlexaFluor 488-conjugated anti-rabbit or anti-rat (all Live Technologies, Karlsruhe, Germany) secondary antibody. Negative controls were performed by omitting the primary antibodies. Nuclei were stained with 4',6-diamidino-2-phenylindole (DAPI) (Roche, Grenzach-Whylen, Germany). LSEC were treated with CPZ (30 μM), filipin (15 nM), nystatin (10 nM) or AMD3100 (10 μM) added to the lower chamber for 10 min (CPZ, nystatin, filipin) or 60 min (AMD3100). LSEC were washed, followed by chemokine incubation (60 min) as described above. Images were taken with an LSM 510 Meta confocal laser scanning microscope (Carl Zeiss MicroImaging, Heidelberg, Germany).

### Immunohistochemistry and immunofluorescence

To quantify T-cell infiltration, paraffin-embedded liver sections were stained with rabbit anti-CD3 antibody (N1580; Dako, Hamburg, Germany) followed by anti-rabbit secondary antibody (Dianova, Hamburg, Germany) and the Streptavidin-Alkaline Phosphatase Kit (Dako) using Fast Red as chromogen. All CD3+ cells per high power field (hpf) and three hpf per liver sample were counted. For immunofluorescence, shock-frozen liver tissue was cut into 4 μm sections and stained with rat anti-CD146 antibody followed by AlexaFluor 488-labeled anti-rat secondary antibody (Dianova). Subsequently, liver sections were stained with rabbit anti-CXCL9 (Santa Cruz Biotechnology) followed by AlexaFluor 455-labeled anti-rabbit secondary antibody (Dianova). Negative controls were performed by omitting the primary antibodies. Images were taken with an AxioImager Z1 microscope and processed with Axiovision software (Carl Zeiss MicroImaging).

### Hepatitis induction and CPZ treatment

Concanavalin (Con) A (20 mg/kg; Sigma-Aldrich) was injected intravenously to induce T cell-mediated hepatitis in C57BL/6 mice. CPZ (3 mg/kg) was intraperitoneally administered 60 min after Con A-treatment. Blood was drawn from individual mice to monitor progression of hepatitis. Liver injury was quantified by automated measurement of plasma activities of alanin transaminase (ALT) using a Roche modular analyzer.

### *In vivo* homing assay

Mice received CPZ 7 h after Con A treatment. Radioactively labeled CD4+ T cells (1x106; 20 μCi 51Cr; GE Healthcare, Munich, Germany) were intravenously injected 120 min after administration of CPZ. After 60 min migration time, the radioactivity of the liver and the remaining body was counted using a Wizard gamma counter (Wallac, Turku, Finland). The percentage of organ-specific radioactivity in relation to the total recovered radioactivity reflected the percentage of cells that have migrated into the respective organ [28].

### Data analysis

Data were analyzed and statistical significance was determined using the GraphPad Prism software (San Diego, CA). Statistical comparison was carried out using the nonparametric two-tailed Mann-Whitney test.

## Results

### Hepatic expression of the inflammatory chemokines CXCL9 and CXCL10 is strongly increased during T cell-mediated hepatitis

**Fig 1:**
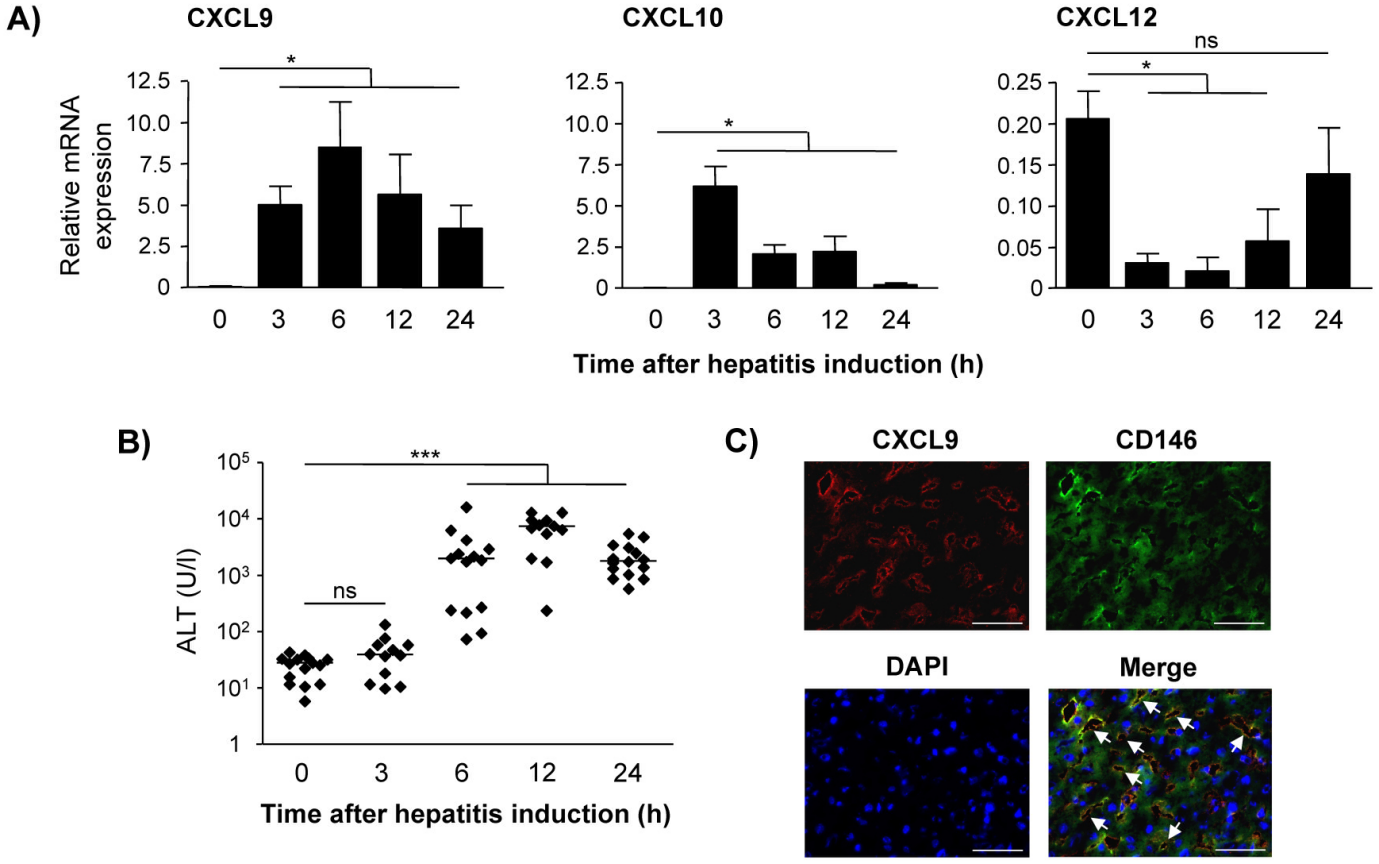
Hepatic chemokine expression during T cell-mediated hepatitis. Con A was intravenously injected into C57BL/6 mice. (A) Quantitative RT-PCR analysis of CXCL9, CXCL10 and CXCL12 mRNA expression in liver tissue of healthy or Con A-treated mice was performed. The chemokine expression was quantified in relation to GAPDH as a housekeeping gene. Mean values ± SD of 4–5 mice per group are shown. (B) Plasma ALT levels were determined at indicated time points. Medians of three independent experiments with 4–5 mice per group are shown. (C) Liver samples were stained with anti-CD146 and anti-CXCL9 antibody 12 h after hepatitis induction. Nuclei were stained with DAPI. Arrows indicate co-localization of CXCL9 and CD146. Bars represent 50 μm. Images are representative of three independent experiments. * p< 0.05; *** p< 0.001; ns, not significant.

*In situ* production, transport and presentation of chemokines in the liver are crucial for the hepatic recruitment of circulating T cells [10,29,30]. In order to identify chemokines induced in the inflamed liver, we used the model of Con A-induced T cell-mediated hepatitis and analyzed hepatic expression of inflammatory and homeostatic chemokines. The mRNA expression of inflammatory CXCL9 and CXCL10 was significantly increased 3 h after induction of hepatitis (Fig 1A), a time point well before liver damage became detectable by plasma ALT levels (Fig 1B). In contrast, the mRNA expression of the homeostatic chemokine CXCL12 was significantly reduced at the same time point. CXCL9 and CXCL10 mRNA expression decreased over time whereas CXCL12 recovered to its initial levels within 24 h after hepatitis induction (Fig 1A). By using immunofluorescence analysis, we detected CXCL9 in the inflamed liver tissue. It particularly co-localized with CD146 demonstrating that CXCL9 was associated with LSEC lining the liver sinusoids (Fig 1C).

### LSEC express and internalize homeostatic and inflammatory chemokines

**Fig 2:**
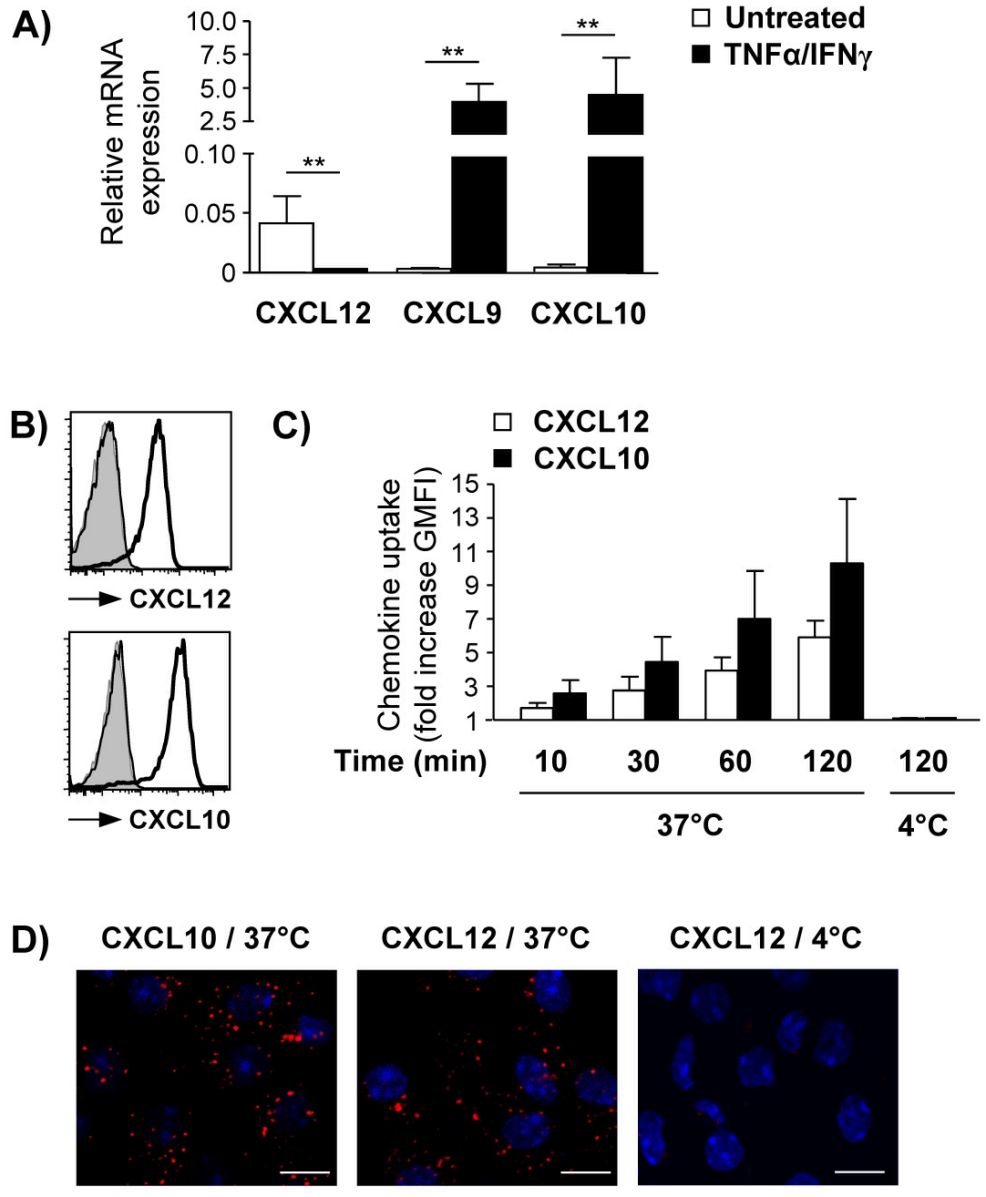
Chemokine expression and internalization by LSEC. (A) Chemokine mRNA expression was quantified in resting and TNF-α/IFN-γ activated LSEC in relation to GAPDH. (B) LSEC layer were incubated with fluorochrome-labeled CXCL10 or CXCL12 at 37°C or 4°C. Histograms show chemokine uptake determined by flow cytometry. Filled graph, no chemokine at 37°C; thin line overlapping with filled graph, chemokine incubation at 4°C; bold line, chemokine incubation at 37°C. (C) Diagram shows fold increase of geometric mean fluorescence intensity (GMFI) of LSEC incubated with chemokine in relation to GMFI without chemokine incubation at 37°C. Mean values ± SD of 4 independent experiments are shown. (D) LSEC were incubated with fluorochrome-labeled CXCL10 or CXCL12 added to the lower chamber of the transwell for 30 min. Nuclei were stained with DAPI. Representative images of three independent experiments are shown. Bars represent 10 μm. Mean values ± SD of 2–4 independent experiments are shown. ** p< 0.01.

Endothelial chemokine expression or internalization of chemokines produced by other liver cells can account for the presence of chemokines in LSEC. We studied the expression of homeostatic and inflammatory chemokines in LSEC by quantitative RT-PCR analysis. In resting LSEC, we detected expression of CXCL12 mRNA but not of CXCL9 and CXCL10 mRNA. Establishing inflammatory conditions *in vitro*, LSEC were activated with TNF-α and IFN-γ. Activated LSEC markedly down-regulated CXCL12 whereas CXCL9 and CXCL10 expression was strongly increased (Fig 2A).

To analyze chemokine uptake, we incubated LSEC with fluorochrome-labeled CXCL12 or CXCL10 and determined the intracellular fluorescence intensity by flow cytometry (Fig 2B). We showed a time-dependent internalization of both chemokines at 37°C that was absent at 4°C (Fig 2C) indicating that chemokine internalisation by LSEC was an active, energy-dependent process. In addition, LSEC cultured on transwell membranes were incubated with fluorochrom-labeled CXCL12 or CXCL10 present in the lower chamber of the transwell and we visualized basolateral internalization and localization of both chemokines in vesicle-like structures in LSEC (Fig 2D).

In summary, LSEC expressed and internalized homeostatic as well as inflammatory chemokines.

### LSEC internalize CXCL12 via CXCR4-mediated endocytosis

**Fig 3:**
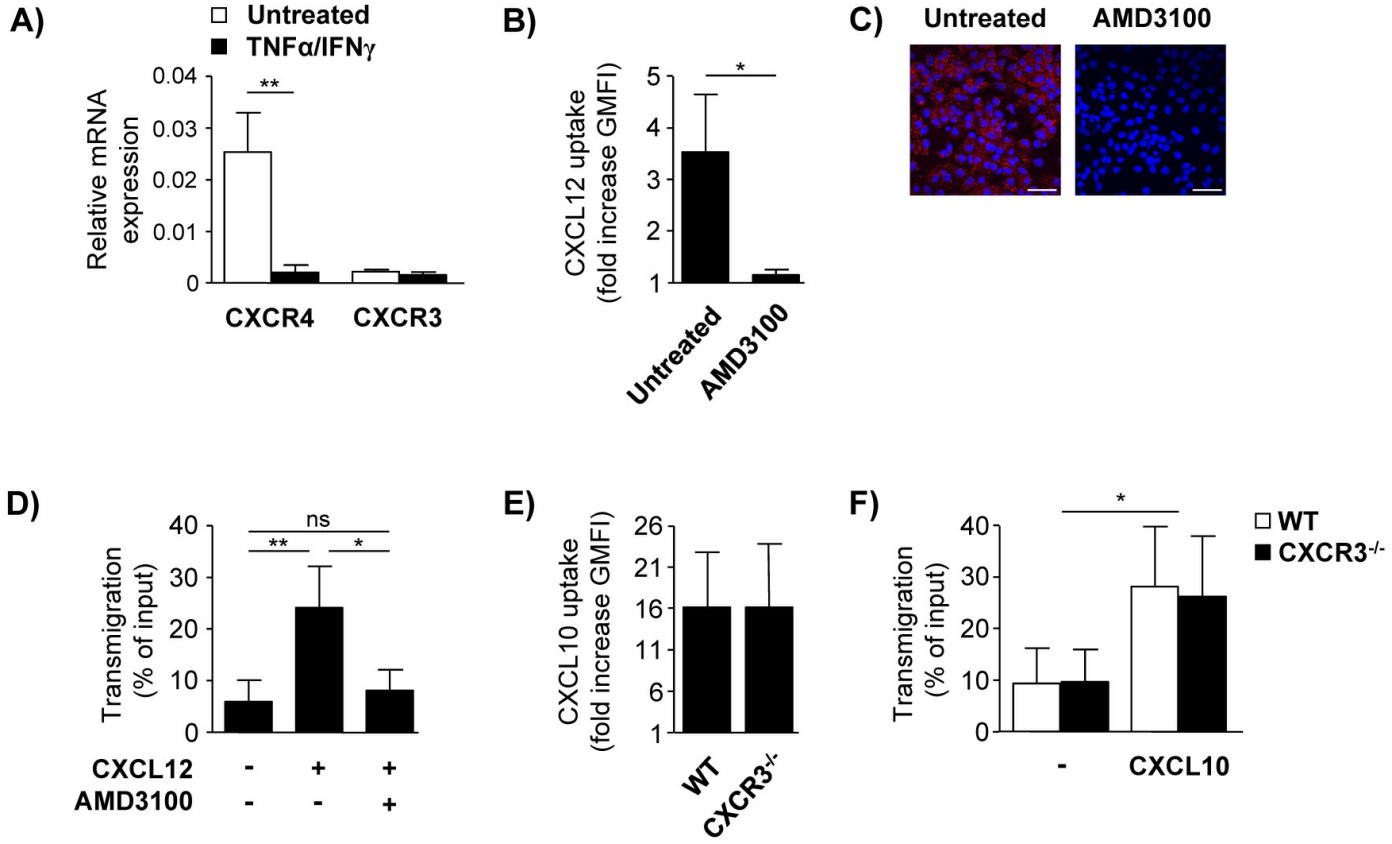
Receptor-mediated chemokine internalization by LSEC. (A) Expression of CXCR3 and CXCR4 mRNA in resting and *in vitro* activated LSEC was quantified in relation to GAPDH. (B) LSEC were treated with AMD3100 prior to incubation with fluorochrome-labeled CXCL12 for 60 min and analyzed by flow cytometry. (C) LSEC were treated with AMD3100 present in the lower chamber of the transwell prior to incubation with AlexaFluor 647-labeled CXCL12 added to the lower chamber for 60 min. Nuclei were stained with DAPI. Representative images of three independent experiments are shown. Bars represent 20 μm. (D) LSEC were treated with AMD3100 present in the lower chamber of the transwell prior pre-incubation with CXCL12 added to the lower chamber for 120 min. After removal of unbound chemokine, transmigration assays with total CD4^+^ T cells were performed. (E) LSEC from CXCR3^-/-^ mice were incubated with fluorochrome-labeled CXCL10 for 120 min. (F) CXCR3-deficient LSEC were pre-incubated with CXCL10 present in the lower chamber of the transwell for 120 min. Transmigration assays with effector/memory CD4^+^ T cells were performed. Mean values ± SD of 2–4 independent experiments are shown. * p< 0.05; ** p< 0.01; ns, not significant.

Chemokines are bound by their corresponding receptors and we asked whether LSEC take up chemokines by receptor-mediated endocytosis. Resting LSEC expressed CXCR4, the receptor for CXCL12, which was strongly decreased after treatment with TNF-α and IFN-γ. In contrast, levels of CXCR3 mRNA were very low in resting LSEC and not increased under inflammatory conditions (Fig 3A). Blockage of CXCR4 by the specific antagonist AMD3100 inhibited CXCL12 internalisation in LSEC (Fig 3B and 3C). We further analyzed whether inhibition of basolateral CXCL12 uptake in LSEC affect CXCL12-dependent transmigration of CD4+ T cells across LSEC. To avoid direct inhibitory effects of AMD3100 on CD4+ T cells, we sequentially pre-incubated LSEC layers with the CXCR4 inhibitor and CXCL12 applied to the lower chamber of the transwell. After removal of unbound chemokine, transmigration assays with CD4+ T cells were performed. Basolateral pre-incubation of LSEC with CXCL12 led to significantly increased CD4+ T-cell transmigration and blockage of endothelial CXCR4 inhibited this process (Fig 3D). Corresponding to the very low expression of CXCR3 by LSEC, neither internalisation of CXCL10 by LSEC from CXCR3-deficient mice (Fig 3E) nor CXCL10-dependent transmigration of effector/memory CD4+ T cells across CXCR3-/- LSEC layer was affected compared to wild type (WT) LSEC (Fig 3F).

These data demonstrate that LSEC internalized CXCL12 via CXCR4-mediated endocytosis leading to enhanced CD4+ T-cell transmigration whereas CXCL10 facilitated transmigration of effector/memory CD4+ T cells but its endothelial uptake was independent of the specific receptor.

### LSEC internalize chemokines in clathrin-coated vesicles

**Fig 4:**
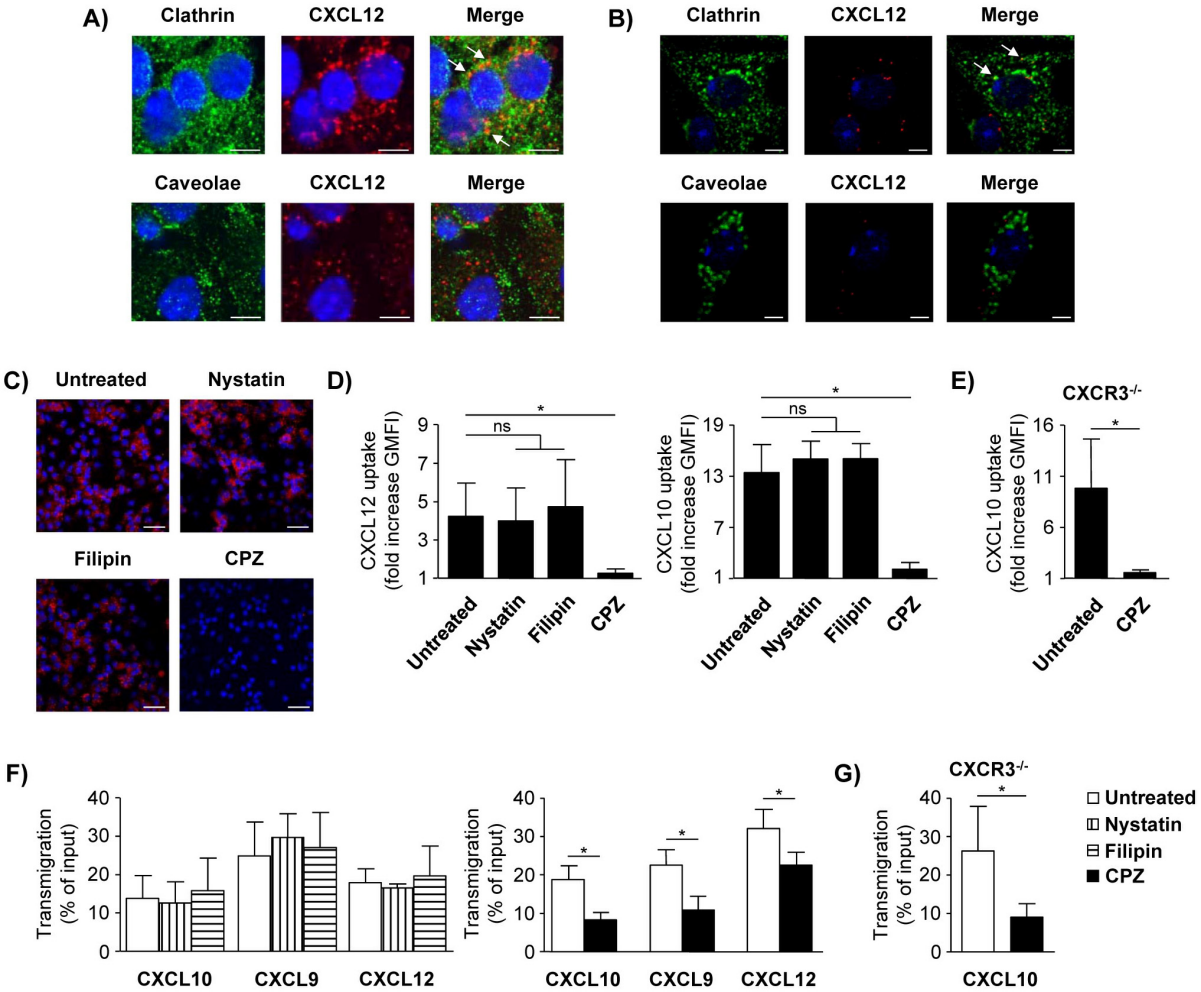
Interference of LSEC-mediated chemokine supply by clathrin and caveolae inhibitors. (A) LSEC were incubated with AlexaFluor 647-labeled CXCL12 added to the lower chamber of the transwell for 10 min. LSEC were stained with anti-clathrin or anti-caveolin-1 antibody. Arrows indicate co-localization. Representative images of 2–4 independent experiments are shown. (B) Clathrin-LCa-EYFP (clathrin) or CAV1-mEGFP (caveolin-1) transfected LSEC were incubated with CXCL12-AlexaFluor 647 added to the lower chamber for 10 min. LSEC were stained with anti-GFP antibody. Nuclei were stained with DAPI. Bars represent 5 μm. (C) LSEC were treated with nystatin, filipin or CPZ present in the lower chamber of the transwell prior to incubation with CXCL12-AlexaFluor 647 added to the lower chamber for 60 min. Representative images of three independent experiments are shown. Bars represent 20 μm. (D, E) LSEC from (D) WT or (E) CXCR3^-/-^ mice were treated with the indicated substances prior to incubation with fluorochrome-labeled CXCL12 or CXCL10 for 60 min. Bar graphs show GMFI fold increase of LSEC incubated with chemokine compared to GMFI without chemokine incubation. (F, G) LSEC layer from (F) WT or (G) CXCR3^-/-^ mice were treated with the indicated substances present in the lower chamber of the transwell. After washing, LSEC were incubated with the chemokines added to the lower chamber for 120 min. After removal of unbound chemokine, transmigration assays with total CD4^+^ T cells (CXCL12) and effector/memory CD4^+^ T cells (CXCL10, CXCL9) were performed. Mean values ± SD from 2–4 individual experiments are shown. * p< 0.05; ns, not significant.

Clathrin-dependent endocytosis and caveolae are the two major pathways mediating internalization and sorting of extracellular molecules [31]. To define the pathway by which LSEC internalize chemokines, LSEC were basolaterally incubated with CXCL12 and stained for clathrin or caveolin-1. CXCL12 co-localized with clathrin but not with caveolin-1 (Fig 4A). To confirm these findings, LSEC were transfected for the transient overexpression of tagged clathrin or caveolin-1 and stained with anti-GFP. We again showed co-localization of CXCL12 with clathrin but not with caveolin-1 (Fig 4B).

LSEC were treated with the caveolae-specific inhibitors nystatin or filipin or with the specific clathrin inhibitor CPZ prior to basolateral chemokine incubation. Neither nystatin nor filipin affected the uptake of CXCL12 or CXCL10 in LSEC. In contrast, CPZ-treated LSEC were strongly impaired in their ability to internalize both chemokines (Fig 4C and 4D). In addition, the inhibitory effect of CPZ on endothelial chemokine internalization was also shown for CXCL10 uptake in CXCR3-/- LSEC (Fig 4E). In line with these data, basolateral treatment of LSEC layers with the caveolae-specific inhibitors prior to chemokine incubation did not alter chemokine-dependent CD4+ T-cell transmigration. However, CPZ treatment significantly decreased CXCL9- and CXCL10-dependent transmigration of effector/memory CD4+ T cells as well as CXCL12-triggered transmigration of CD4+ T cells across LSEC layer (Fig 4F). The CXCL10-dependent transmigration of effector/memory CD4+ T cells across CXCR3-/- LSEC layer was also strongly reduced after treatment with CPZ (Fig 4G).

In summary, the clathrin pathway proved crucial in LSEC-mediated chemokine transfer to CD4+ T cells.

### LSEC transfer internalized chemokines to early endosomes but not to recycling endosomes or lysosomes

**Fig 5:**
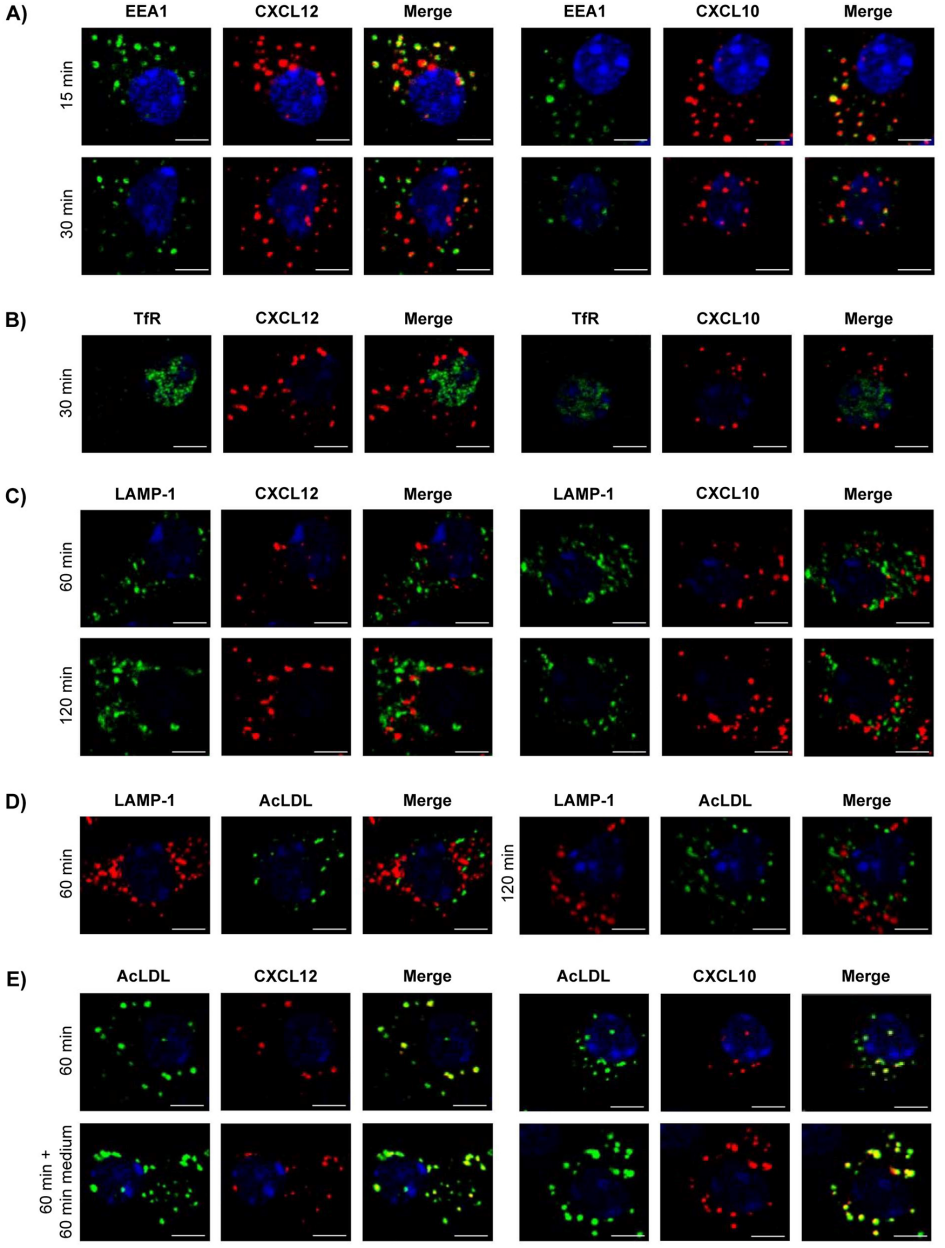
Co-localization of CXCL12 and CXCL10 with components of the endocytic pathway. LSEC were incubated with AlexaFluor 647-labeled CXCL12 or CXCL10 present in the lower chamber of the transwell. LSEC were stained with (A) anti-EEA1, (B) anti-TfR or (C) anti-LAMP-1 antibody. (D) LSEC were incubated with AlexaFluor 488-labeled AcLDL present in the lower chamber of the transwell and stained with anti-LAMP-1 antibody. (E) LSEC were incubated with AlexaFluor 647-labeled chemokine and AcLDL-AlexaFluor 488 present in the lower chamber of the transwell. Representative images of two independent experiments are shown. Bars represent 5 μm.

To further determine molecular mechanisms involved in endothelial chemokine transport, we performed co-localization studies of chemokines and components of the endocytic pathway. We identified early endosomes and recycling endosomes by staining with antibodies specific for EEA1 [32] and TfR [33], respectively. Lysosomes were identified by staining for LAMP-1 [34]. LSEC were basolaterally incubated with CXCL12 or CXCL10 for indicated time points. After 15 min, both chemokines showed co-localization with EEA1 that was strongly decreased after 30 min (Fig 5A, Table 1). At this time point, neither CXCL10 nor CXCL12 co-localized with TfR (Fig 5B, Table 2). After 60 min, CXCL10 and CXCL12 did not co-localize with LAMP-1 and co-localization was also not found after 120 min of incubation (Fig 5C, Table 3).

**Table 1:** Co-localization of CXCL10 and CXCL12 with EEA1 in LSEC.

Chemokine	Time (min)	Cell #	# vesicles per cell		Co-localization with EEA1 (%)
			red	yellow	
CXCL10	15	91	10 (5; 15)	7 (3.5; 10)	75 (61.8; 88.6)
	30	85	10 (6; 13)	4 (2; 6)	38.9 (21.1; 55.6)
CXCL12	15	106	8 (6; 13)	6 (4; 9)	75 (66.7; 87.5)
	30	105	12 (7; 17)	5 (3; 8)	43.5 (28.6; 59.1)

LSEC were incubated with AlexaFluor 647-labeled chemokine (red) present in the lower chamber of the transwell for 15 min or 30 min. Cells were stained with anti-EEA1 antibody (green). Red and yellow vesicles were counted per cell and co-localization was calculated as % of yellow vesicles within red ones. Medians (1. quartile; 3. quartile) of two independent experiments are given.

**Table 2:** Co-localization of CXCL10 and CXCL12 with TfR in LSEC.

Chemokine	Time (min)	Cell #	# vesicles per cell		Co-localization with TfR (%)
			red	yellow	
CXCL10	30	97	11 (7; 15)	0 (0; 1)	0 (0; 9.5)
CXCL12	30	102	10 (7.5; 15)	0 (0; 1)	0 (0; 10)

LSEC were incubated with AlexaFluor 647-labeled chemokine (red) present in the lower chamber of the transwell for 30 min. Cells were stained with anti-TfR antibody (green). Medians (1. quartile; 3. quartile) of two independent experiments are given.

**Table 3:** Co-localization of CXCL10, CXCL12 and AcLDL with LAMP-1 in LSEC.

Reagent	Time (min)	Cell #	# vesicles per cell		Co-localization with LAMP-1 (%)
			red/green	yellow	
CXCL10	60	96	12 (7.8; 18)	0 (0; 1)	0 (0; 6.4)
	120	73	10 (6; 20)	0 (0; 1)	0 (0; 6.3)
CXCL12	60	97	13 (7; 17)	0 (0; 1)	0 (0; 5.9)
	120	79	14 (9; 20)	0 (0; 1)	0 (0; 6.9)
AcLDL	60	37	18 (11; 24)	0 (0; 1)	0 (0; 3.3)
	120	28	16.5 (10; 20)	0 (0; 1)	0 (0; 5.9)

LSEC were incubated with an AlexaFluor 647-labeled chemokine (red) or AlexaFluor 488-labeled AcLDL (green) present in the lower chamber of the transwell for 60 min or 120 min. Cells were stained with anti-LAMP-1 antibody (green in combination with CXCL10 or CXCL12; red in combination with AcLDL). Medians (1. quartile; 3. quartile) of two independent experiments are given.

Interestingly, basolaterally internalized AcLDL, a protein that usually ends up in the lysosome [35], also did not co-localize with LAMP-1 in LSEC after 60 min and 120 min of incubation (Fig 5D, Table 3). In addition, both chemokines showed a strong co-localization with AcLDL after 60 min incubation time and also after incubation in medium for another hour pointing to a shared intracellular transport pathway (Fig 5E, Table 4).

**Table 4:** Co-localization of CXCL10 and CXCL12 with AcLDL in LSEC.

Chemokine	Time (min)	Cell #	# vesicles per cell		Co-localization with AcLDL (%)
			red	yellow	
CXCL10	60	87	8 (5.5; 12.5)	7 (5; 11.5)	90.9 (81.7; 100)
	60 + 60	94	10 (7; 14)	10 (6.3; 13.8)	100 (91.1; 100)
CXCL12	60	81	7 (5; 11)	7 (5; 10)	100 (92.9; 100)
	60 + 60	83	9 (6; 12)	8 (5.5; 11)	100 (88.9; 100)

LSEC were incubated with AlexaFluor 647-labeled chemokine (red) and AlexaFluor 488-labeled AcLDL (green) present in the lower chamber of the transwell for 60 min or for additional 60 min after removal of chemokine and AcLDL. Medians (1. quartile; 3. quartile) of two independent experiments are given.

### Recruitment of CD4+ T cells into the inflamed liver is decreased after administration of CPZ

During liver inflammation, the rate of T-cell recruitment via the sinusoidal endothelium strongly increases [36]. Having shown *in vitro* that CPZ affects endothelial chemokine transfer leading to reduced transmigration of CD4+ T cells across LSEC layer we asked whether CPZ also influences CD4+ T-cell recruitment to the inflamed liver.

**Fig 6:**
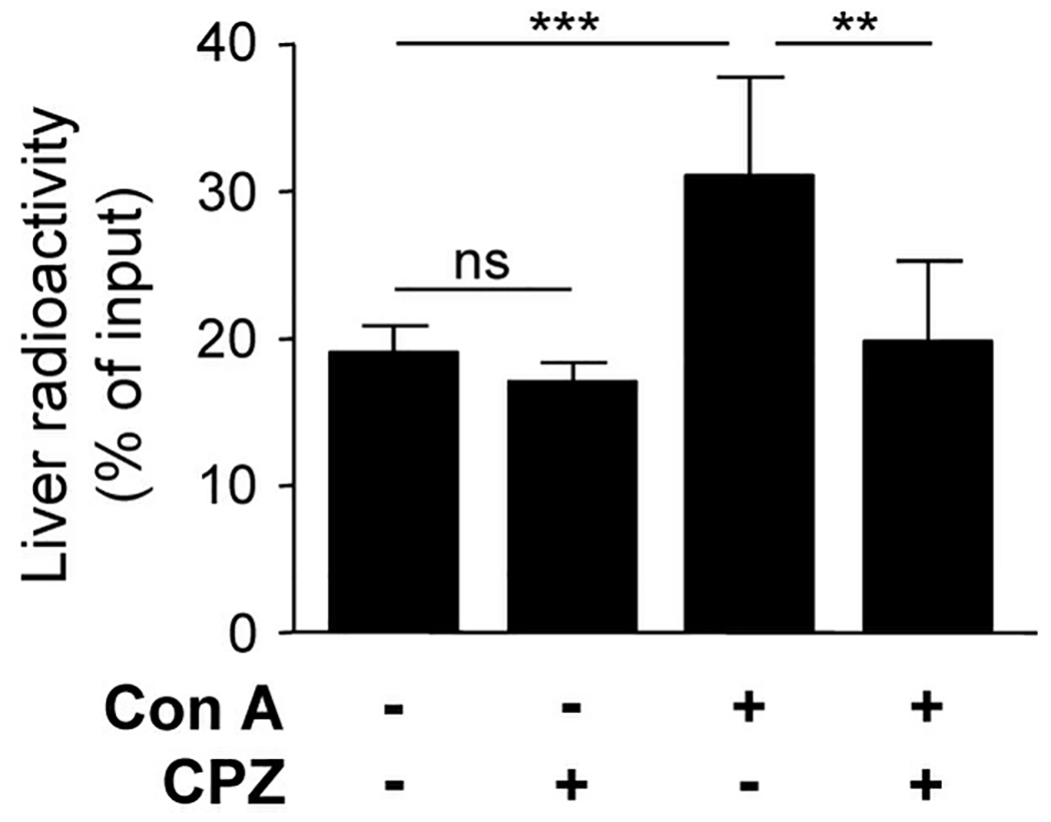
Migration of CD4^+^ T cells into the inflamed and healthy liver after administration of CPZ. C57BL/6 mice were treated with Con A and received CPZ 7 h after hepatitis induction. Healthy mice also received CPZ. Radioactively labeled total CD4^+^ T cells were intravenously transferred into mice 120 min after administration of CPZ. Liver-specific radioactivity in relation to total radioactivity of the body was determined after 60 min migration time. Mean values ± SD of 4 independent experiments with three mice per group are shown. ** p< 0.01; *** p< 0.001; ns, not significant.

To induce hepatic inflammation, mice were treated with Con A and received CPZ 7 h later, a time point when hepatitis was established (see Fig 1B). Radioactively labeled CD4+ T cells were transferred into the mice and their immigration into the liver was assessed by an *in vivo* homing assay. In mice that received Con A, recruitment of CD4+ T cells to the inflamed livers was significantly enhanced compared to livers of healthy mice. Strikingly, despite liver inflammation, migration of CD4+ T cells into the liver was significantly reduced if mice received CPZ in addition to Con A. CPZ itself did not influence hepatic migration of transferred CD4+ T cells in healthy mice (Fig 6).

### Accumulation of CXCR3+ CD4+ T cells in the inflamed liver is reduced after administration of CPZ

**Fig 7:**
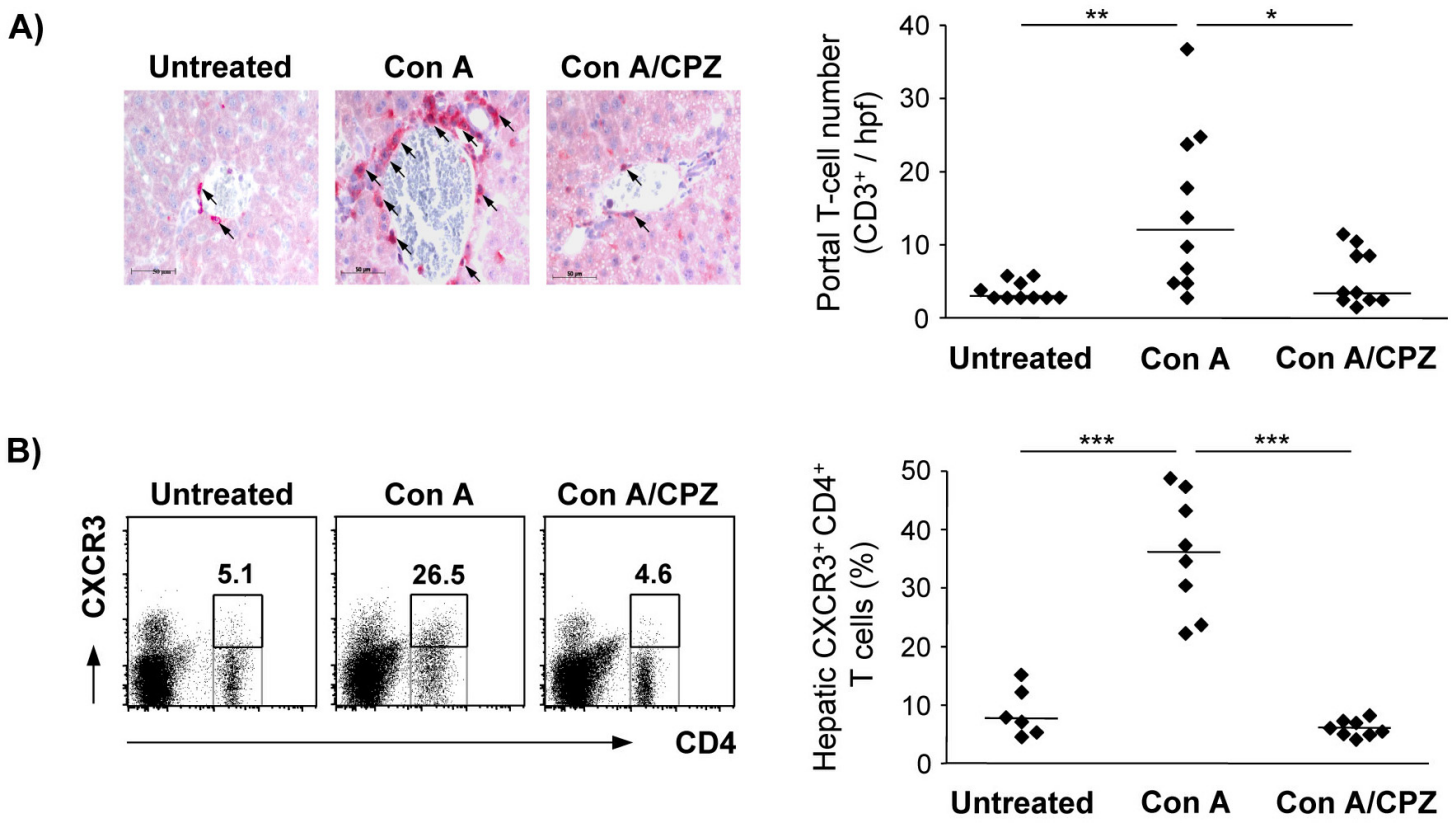
Hepatic accumulation of CXCR3^+^ CD4^+^ T cells during T cell-mediated hepatitis after administration of CPZ. Mice were treated with Con A and received CPZ 60 min after hepatitis induction. (A) Liver samples were stained with anti-CD3 antibody 24 h after hepatitis induction. Portal CD3^+^ T-cell numbers were counted per hpf. Arrows indicate T cells. Representative images and medians of two independent experiments with 5 mice per group. Bars represent 50 μm. (B) NPC were isolated 24 h after hepatitis induction, stained with anti-CD4 and anti-CXCR3 antibody and analyzed by flow cytometry. Representative dot plots and medians of two independent experiments with 3–4 mice per group. * p< 0.05; ** p< 0.01; *** p< 0.001.

During autoimmune hepatitis, activated T cells accumulate within the liver. We again used the model of Con A-induced T cell-mediated hepatitis to study the effect of interfering with the clathrin pathway on hepatic T-cell infiltration during liver inflammation. Mice were treated with Con A and received CPZ 60 min after hepatitis induction, a time point when migration of activated T cells to the liver has not yet taken place. One day after hepatitis induction, liver tissue was stained with an anti-CD3 antibody to identify T cells. We showed a strong hepatic accumulation of CD3+ T cells around the portal areas during liver inflammation. This T-cell accumulation was significantly decreased after administration of CPZ (Fig 7A). Arguing against direct effects of CPZ on T-cell activation *in vivo*, we detected similar expression of the early activation marker CD69 and the inflammatory cytokine IFN-γ in hepatic CD90.2+ total T cells as well as CD4+ T cells of mice that received CPZ and Con A compared to those of only Con A-treated mice (S1 Fig).

We also tested the effect of CPZ on chemokine-dependent T-cell infiltration during liver inflammation. We quantified the fraction of hepatic CD4+ T cells expressing CXCR3, the receptor for CXCL9 and CXCL10 that were strongly up-regulated at an early stage of Con A-induced hepatitis (see Fig 1A). The percentage of CXCR3+ CD4+ T cells significantly increased in the inflamed livers of Con A-treated mice compared to those of healthy mice. Interestingly, in mice that received CPZ the percentage of hepatic CXCR3+ CD4+ T cells was significantly reduced (Fig 7B) further supporting the finding that inflammation-induced T-cell migration to the liver is diminished after administration of a clathrin inhibitor *in vivo*.

## Discussion

T-cell migration into the liver tissue is a major factor determining the pathogenesis of hepatic inflammation. Hepatic expression of CXCR3 ligands is strongly induced in many inflammatory liver diseases [10–12,37] and infiltrating effector T cells express high levels of CXCR3 [38–41]. During liver inflammation, CXCR3 ligands have been shown to be expressed by various liver resident cells inclusive LSEC, hepatocytes, stellate cells, and cholangiocytes [30,42–45]. In Con A-induced hepatitis, we found CXCL9 in the inflamed liver to be closely associated with LSEC, which represent the first contact site for T-cell immigration into the liver. Under inflammatory conditions *in vitro*, LSEC expressed CXCL9 and CXCL10 further emphasizing the finding that LSEC contribute to the reservoir of chemokines that are expressed during liver inflammation.

Beside chemokine expression LSEC were able to actively internalize basolateral chemokines which may be secreted by other cell types of the subadjacent liver tissue. Perivascularly expressed chemokines can be transported across the endothelium [42,46–50] and immobilized on the endothelial glycocalyx [42,51,52] to support localized lymphocyte recruitment. We have previously shown that LSEC transfer basolaterally internalized chemokines to CD4+ T cells thereby enhancing T-cell transmigration [18]. What structures are involved in the endothelial trafficking of chemokines? Chemokine receptors mainly regulate leukocyte migration but have also been identified on endothelia [53]. The functional outcome of CXCR4 expression differs between endothelial cells and T cells. CXCR4+ T cells do not efficiently take up CXCL12 but are highly motile in response to this chemokine whereas tissue-anchored CXCR4+ bone marrow endothelial cells regulate CXCL12 availability by transcytosis [47]. Correspondingly, we here demonstrated that the blockage of LSEC-expressed CXCR4 inhibited CXCL12 internalization and CXCL12-dependent CD4+ T-cell transmigration strongly indicating a role of this chemokine receptor in LSEC-mediated chemokine transport to CD4+ T cells. In homeostasis, CXCL12 was constitutively expressed in liver tissue and we assumed that the LSEC-mediated transfer of CXCL12 from the tissue to the blood-endothelial interface is involved in T-cell migration as part of the hepatic immune surveillance. In acute T cell-induced hepatitis, CXCL12 expression was markedly down-regulated suggesting that this chemokine did not mediate hepatic T-cell recruitment during Con A-induced liver inflammation. However, CXCL12-triggered infiltration of CXCR4+ lymphocytes has been described during hepatitis C and B virus infection [54] thus, CXCR4-dependent endothelial CXCL12 transport may also play a role in the pathogenesis of chronic liver infections.

In contrast to endothelial CXCL12 internalization which strictly depended on CXCR4, LSEC did not express CXCR3 and CXCL10 was internalized independently of its receptor. Another study reported expression of CXCR3 by dermal and lung microvessel endothelium but also in this case the uptake of CXCL10 was not receptor dependent [50]. Together with the fact that CXCR3 expression in LSEC was also not induced under inflammatory conditions, where endothelial transport of inflammatory chemokines may be of particular importance, our data emphasize the assumption that mechanisms relating to CXCL9/10-induced signal transduction in leukocytes are different from those used for endothelial trafficking. CXCR4 mediates both CXCL12-triggered signal transduction in motile leukocytes as well as transport of CXCL12 across an endothelium. In contrast, the main function of CXCR3 seems to be signal transduction in leukocytes in order to induce their recruitment to sites of inflammation whereas endothelial transport of its ligands is mediated by other, so far not identified molecular mechanisms. The non-signaling chemokine receptor Duffy antigen receptor is expressed on endothelial cells and erythrocytes and binds chemokines of the CC and CXC family but not CXCL12. It has been shown that the functional outcome of chemokine binding by Duffy antigen receptor for chemokines is transcytosis [55,56] and it would be conceivable that this interceptor is involved in the transport of the CXCR3 ligands across LSEC. Another study also described a receptor-independent mechanism by which glycosaminoglycans mediated endothelial transcytosis of inflammatory chemokines [57]. In our model of acute T cell-mediated hepatitis, fast up-regulation of CXCL9 and CXCL10 in the liver tissue preceded parenchymal accumulation of effector T cells. It is conceivable that enhanced local production and LSEC-mediated transport and presentation of the CXCR3 ligands supported hepatic recruitment of CXCR3+ effector CD4+ T cells triggering inflammation as well as CXCR3+ regulatory CD4+ T cells necessary to suppress hepatitis [58] and the balance of the cells recruited will determine the outcome of the liver disease.

Chemokine receptors undergo ligand-induced internalization through clathrin-mediated endocytosis [47,59,60]. CPZ causes disruption of clathrin-coated vesicles [25]. Focussing on this aspect of CPZ action, treatment of LSEC with CPZ inhibited internalization of both CXCL12 and CXCL10 and significantly decreased chemokine-dependent transmigration of CD4+ T cells across the LSEC layer. The clathrin-dependent internalization and presentation of CXCL9 and CXCL10 in CXCR3-/- LSEC further support the assumption that a so far not identified receptor is involved in the transport of the CXCR3 ligands across endothelial cells.

Several intracellular pathways regulate the fate of proteins after their internalization. Co-localization studies in LSEC showed that internalized chemokines were transferred to clathrin-coated vesicles but not to caveolae. Corresponding to this finding, inhibition of caveolae in LSEC neither affected chemokine internalization nor chemokine-dependent CD4+ T-cell transmigration. Endothelial chemokine transport has been described to be mediated by either caveolae or clathrin-coated vesicles dependent on the type of endothelium [47,49]. We excluded caveolae and demonstrated that LSEC used the clathrin pathway for chemokine transport. We also showed that CXCL12 and CXCL10 were transferred to EEA1+ early endosomes shortly after internalization, an endocytic compartment downstream of the clathrin-mediated uptake. Both chemokines left the early endosomes again but neither co-localized with LAMP-1 nor with TfR suggesting that LSEC did not primarily transfer basolaterally internalized chemokines to the lysosomes for degradation or to the recycling endosomes to return them back to the basolateral plasma membrane. Moreover, AcLDL, a protein that is internalized via scavenger receptors and usually degraded after internalization, was also not transferred to the lysosomes and co-localized with the chemokines in LSEC. On the basis of these data, we assume that the liver sinusoidal endothelium mainly transported perivascularly produced proteins like chemokines to the blood-endothelial interface. The striking co-localization of chemokines and AcLDL in LSEC suggests that scavenger receptors might be involved in endothelial chemokine supply. We found no evidence that the type of chemokine to be transported through the endothelial cell layer is selected. Thus, the pattern of chemokines presented by LSEC seems to depend on the chemokine pool produced by liver resident cells which differs under homeostatic and inflammatory conditions.

Having demonstrated that inhibition of the clathrin pathway by CPZ reduced the chemokine-dependent transmigration of CD4+ T cells *in vitro* we also analyzed whether CPZ affects T-cell migration into the liver *in vivo*. We showed that the strongly increased recruitment of transferred CD4+ T cells into the inflamed liver of Con A-treated mice was abolished after administration of CPZ during hepatitis whereas the clathrin inhibitor did not influence CD4+ T-cell migration to the healthy liver. Moreover, the high frequency of CXCR3+ CD4+ T cells in the inflamed liver tissue was strongly reduced after administration of CPZ. We excluded inhibitory effects of CPZ on T-cell activation *in vivo* and another study also showed that CPZ did not affect inflammatory cytokine production [61] during Con A-induced hepatitis which could be possible reasons for the reduced hepatic infiltration of effector CD4+ T cells. These data demonstrate that the inflammation-induced hepatic recruitment of T cells was especially impaired after treatment with the clathrin inhibitor. CPZ might affect local chemokine production and/or endothelial chemokine transport and presentation thereby attenuating effector T-cell migration into the inflamed liver. Despite potential other effects of CPZ on lymphocyte function, our *in vitro* data suggested CPZ as a suitable model substance for this first study on the impact of clathrin inhibition on the course of liver inflammation.

In summary, the data presented here indicate that the liver sinusoidal endothelium, which acts as an anatomical barrier between the liver parenchyma and the blood circulation, contains an intracellular transport system for the transfer of tissue-expressed chemokines across this barrier in order to induce local recruitment of circulating T cells. We identified CXCR4- and clathrin-dependent intracellular transport mechanisms involved in LSEC-mediated chemokine transport to CD4+ T cells and demonstrated that inhibition of these processes diminished chemokine-dependent CD4+ T-cell transmigration *in vitro*. Our data showing that the inflammation-induced recruitment of effector CD4+ T cells into the inflamed liver can be modified by treatment with a clathrin inhibitor led us to the assumption that endothelial chemokine supply by transcytosis or production plays an important role in the regulation of local inflammation. In this light, it is tempting to speculate that interventions in these processes during liver inflammation could be a target for inhibition of lymphocyte infiltration thereby counteracting immunopathology.

## Supporting Information

S1 Fig Hepatic T-cell activation after administration of CPZ. Mice were treated with Con A and received CPZ 60 min after hepatitis induction. NPC isolated 6 h after Con A treatment and stained for CD4, CD90.2, CD69 and IFN-γ were assessed by flow cytometry. (A) GMFI of CD69 was determined on gated CD90.2^+^ or CD4^+^ T cells. (B) Percentages of CD90.2^+^ and CD4^+^ T cells expressing IFN-γ were detected. Representative plots and mean values ± SD of 2–4 independent experiments with three mice per group are shown. * p< 0.05; ns, not significant.
(TIF)
