# Supplementary figures and images for: Chemokine Transfer by Liver Sinusoidal Endothelial Cells Contributes to the Recruitment of CD4+ T Cells into the Murine Liver
Source: PLoS One. 2015 Jun 8;10(6):e0123867. doi: 10.1371/journal.pone.0123867 (PMC4460118; doi:10.1371/journal.pone.0123867)

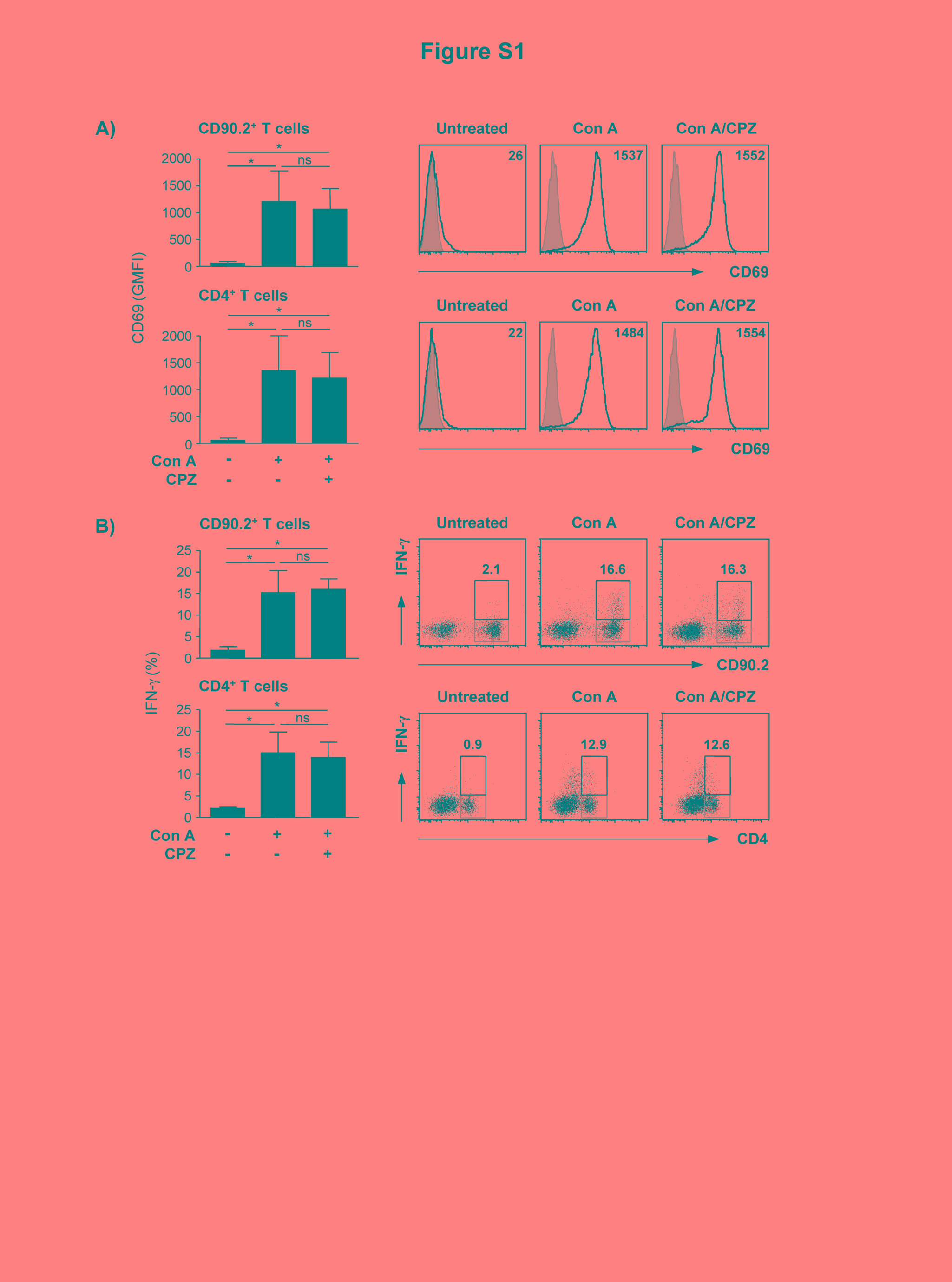

Supplement: S1 Fig — Mice were treated with Con A and received CPZ 60 min after hepatitis induction. NPC isolated 6 h after Con A treatment and stained for CD4, CD90.2, CD69 and IFN-γ were assessed by flow cytometry. (A) GMFI of CD69 was determined on gated CD90.2+ or CD4+ T cells. (B) Percentages of CD90.2+ and CD4+ T cells expressing IFN-γ were detected. Representative plots and mean values ± SD of 2–4 independent experiments with three mice per group are shown. * p< 0.05; ns, not significant. (TIF) [file pone.0123867.s001.tif]
